# Cemiplimab in the treatment of cutaneous squamous cell carcinoma

**DOI:** 10.1080/1750743X.2026.2619658

**Published:** 2026-01-29

**Authors:** Luke S. McLean, Annette M. Lim, Danny Rischin

**Affiliations:** a Department of Medical Oncology, Peter MacCallum Cancer Centre, Melbourne, Victoria, Australia; b Department of Medical Oncology, St Vincent’s Hospital, Fitzroy, Victoria, Australia; c Department of Medicine, The University of Melbourne, Parkville, Australia; d Sir Peter MacCallum Department of Medical Oncology, The University of Melbourne, Parkville, Australia

**Keywords:** Cemiplimab, cutaneous squamous cell carcinoma, immunotherapy, immune checkpoints, neoadjuvant, adjuvant, metastatic

## Abstract

Immune checkpoint inhibitors (ICIs) have significantly transformed the treatment landscape of cutaneous squamous cell carcinoma (CSCC). Cemiplimab, a human IgG4 monoclonal antibody to the programmed death-1 (PD-1) receptor, has shown impressive response rates and durable disease control in patients with advanced CSCC, alongside a manageable safety profile. More recently, cemiplimab has demonstrated promising efficacy in both the adjuvant and neoadjuvant settings, with impressive improvements in disease-free survival and pathological response rates. This review provides a comprehensive overview of the current role of cemiplimab in the management of CSCC, highlighting key clinical trial data and real-world evidence on its efficacy and safety.

## 1. Introduction

Keratinocyte skin cancers, including basal cell carcinoma (BCC) and cutaneous squamous cell carcinoma (CSCC), are the most frequently diagnosed malignancies in Australia and New Zealand. They place a substantial burden on the public health system and are the leading cause of cancer-related hospitalizations with high age-standardized incidence rates of 229.2 per 100,000 and 66.7 per 100,000 in men and women, respectively [1–4]. CSCC accounts for around 20% of all keratinocyte skin cancers and arises from keratinocytes in the epidermis. There are over 2.4 million estimated cases of CSCC diagnosed each year worldwide and around 65,000 deaths [5]. In comparison to BCC, CSCC has a higher propensity for recurrence and metastases, and it is estimated that up to 5% of patients with CSCC develop unresectable locally advanced or metastatic disease [6–8]. Additional factors, such as an aging population, better survival outcomes for patients with hematological malignancies, and an increase in solid organ transplantation have meant the rates of CSCC have increased over time. While most cases of CSCC are curable with surgery and/or radiotherapy, morbidity remains a significant concern due to the multiplicity of lesions and a tendency for involvement of the esthetically sensitive region of the head and neck where surgical intervention can be complex and disfiguring. Immune checkpoint inhibitors (ICI) have transformed the treatment landscape for advanced CSCC, demonstrating high response rates and durable outcomes in both clinical trial settings and real-world populations [9,10]. Real-world data has been of important clinical relevance given the tendency for CSCC to occur in immunocompromised individuals, such as organ transplant recipients, who are often excluded from clinical trials. There is also growing interest in expanding the use of ICIs into neoadjuvant and adjuvant treatment settings [11,12]. Among the ICIs evaluated to date, cemiplimab, a monoclonal antibody targeting programmed death-1 (PD-1), has pioneered changes in CSCC management. This *Drug Review* article will focus on cemiplimab, exploring its clinical development, efficacy, safety profile, and evolving role across different stages of CSCC treatment.

## 2. Overview of the field

### 2.1. Risk factors for cutaneous squamous cell carcinoma (CSCC)

The pathogenesis of CSCC involves a complex interplay of environmental, genetic, and immunological factors, including ultra-violet (UV) radiation, individual characteristics such as skin-type and ethnicity and the presence of a compromised immune system. Chronic cumulative UV-radiation exposure is the most well-established risk factor for CSCC. This contrasts with melanoma and BCC where intermittent sunburn is more strongly implicated [13,14]. Risk significantly increases after approximately 70,000 hours of lifetime sun exposure, and incidence of CSCC rises sharply with age – being 5–10 times more frequent in individuals over 75 years and rare in those under 45 [13,15,16]. Both epidemiological and animal studies support UV radiation as a carcinogen, and it is classified as a Group 1 carcinogen by the International Agency for Research on Cancer [17]. Geographic variation further highlights the role of UV-exposure with higher CSCC rates observed in regions closer to the equator and in predominantly Caucasian populations. For example, CSCC has significantly higher incidence rates in Arizona compared with New Hampshire in the United States and in Queensland compared with Victoria in Australia [18–20]. UV-induced mutations contribute to the high tumor mutational burden (TMB) seen in CSCC with cytidine-to-thymidine transitions (C > T or CC > TT) accounting for more than 80% of these mutations and are the hallmark of the common “UV-signature” detectable in CSCC [21,22].

Immunosuppression is another well-described risk factor for CSCC, with immunocompromised individuals experiencing up to a 100-fold increased risk of its development, accompanied with more aggressive disease defined by higher recurrence rates and poorer treatment outcomes [23–25]. There are many drivers of immunosuppression including hematological malignancy, human immunodeficiency virus (HIV), solid organ transplantation, and medication. Among hematological malignancies, chronic lymphocytic leukemia is frequently observed in advanced CSCC cohorts. This group has an eight-fold increased risk of keratinocyte cancer and a higher risk of death (standardized mortality ratio 17.0, 95% confidence interval (CI) 14.4 – 19.8) [26–29]. HIV infection also increases risk, particularly in those with low CD4 counts or high viral loads [30]. In the solid organ transplant population, CSCC is more common than BCC with a BCC:CSCC ratio of 1:8 [31]. This risk increases with the transplant type (i.e., more common in heart and lung) [32–35] and with the intensity and duration of the immunosuppression [36–38]. Certain immunosuppressive medications such as azathioprine and cyclosporine are linked to increased CSCC risk through photosensitization and interference with p53 function [39,40]. Newer agents like tacrolimus may carry less risk, although findings are mixed [41]. Mycophenolate is considered to carry a lower risk [42,43] and mechanistic target of rapamycin (mTOR) inhibitors such as sirolimus and everolimus have even shown promise in reducing CSCC incidence [44,45]. In one large study of 45,000 kidney transplant recipients, a sirolimus-based immunosuppressive medication regimen was associated with a significant reduction in keratinocyte cancers (HR 0.71, 95% CI 0.60–0.84) [46].

Environmental exposures such as chronic arsenic ingestion, aromatic hydrocarbons, and ionizing radiation have also been implicated in CSCC [47–50]. Chronic inflammation can lead to the development of a molecularly distinct CSCC different to UV-induced disease, with approximately 1% of skin cancers arising in chronically inflamed skin [51]. CSCC has been associated with lichen sclerosis as well as Marjolin’s ulcer where it can arise in scar, ulcers, or even pressure sores [52]. These tumors often lack the classical features of traditional CSCC, such as a high TMB or UV-signature, and are often associated with a more aggressive phenotype and poor prognosis [53,54].

Certain inherited conditions like Fanconi anemia, dyskeratosis congenita, albinism, and epidermodysplasia verruciformis are also linked to an elevated CSCC risk [55–57]. Additionally, beta human papillomavirus (HPV) infection has been implicated in CSCC pathogenesis in both observational and preclinical studies [58–62].

These diverse risk factors underscore the heterogeneity of disease and populations at increased risk for CSCC, with certain groups, such as immunocompromised patients, being particularly susceptible to more aggressive and high-risk forms of the disease.

### 2.2. Local management options for CSCC

Cutaneous squamous cell carcinoma usually presents as a small, indolent primary tumor that is amenable to surgical resection. Groups such as the National Comprehensive Cancer Network and the American Joint Committee on Cancer (AJCC) have identified several prognostic factors to help determine which tumors may be at high risk of recurrence. These factors include the location of the tumor, its depth, size, and differentiation status [63]. Tumors considered low risk are typically small (less than 2 cm), well differentiated and do not demonstrate invasion into nerves, lymphatics, or blood vessels. A high-risk tumor, however, is often larger, has depth of invasion of more than 2–6 mm, is poorly differentiated, and demonstrates invasive features like perineural involvement. For low-risk localized disease, treatment options include surgical excision, curettage, and electrodessication. Cryotherapy and photodynamic therapy are also sometimes used. Surgery remains the treatment of choice for higher risk primary tumors. This can include both standard surgical excision and Mohs micrographic surgery. There are no randomized trials comparing these two approaches and both are considered acceptable options. Early stage localized CSCC has a low recurrence rate of around 4.6% post-surgery [8]. Definitive radiotherapy is another approach with a local recurrence rate of 6.4% and is commonly reserved for those who are not candidates for surgical resection [64–66].

Adjuvant radiotherapy is generally recommended in tumors that exhibit high-risk features such as perineural invasion, positive margins, or nodal involvement [67,68]. However, in the absence of randomized data, the use of adjuvant radiation varies across different regions. The SCC-AFTER phase III randomized controlled trial (ISRCTN54806122) is currently evaluating whether 45–60 gray of adjuvant radiation within 4 months of surgery in resected T2b/3 (Bringham and Women’s Hospital criteria) CSCC is superior to close observation alone. A phase III trial (*n* = 321) demonstrated no benefit when chemotherapy was added to adjuvant radiotherapy in high-risk CSCC, characterized by tumors larger than 5 cm, involvement of intra-parotid lymph nodes, multiple cervical lymph nodes, or nodes exceeding 3 cm or with extracapsular extension [69].

Up to 80% of CSCC cases involve the head and neck region, where aggressive surgery and radiotherapy can significantly affect patient quality of life due to the esthetic and functional importance of this anatomical location [70,71]. This is especially true for tumors extending toward critical structures such as the skull base, temporal bone, nose, or orbit, where curative surgery may cause substantial deformity and morbidity. For example, whilst orbital exenteration can achieve up to 60% 2-year disease-free survival (DFS) for periocular tumors [72], these patients often face chronic pain, anxiety, depression, and social difficulties due to the loss of the eye [73]. Similarly, maxillectomy can impair speech, swallowing, and chewing functions and lateral temporal bone resection may also result in conductive hearing loss [74,75]. Radiotherapy can also cause fibrosis, lymphedema, and skin necrosis in this sensitive region. Consequently, improving quality of life by minimizing morbidity associated with curative treatments without compromising survival remains a key research focus in locally advanced CSCC. This has driven increasing interest in the use of ICIs, particularly cemiplimab, as a strategy to reduce treatment-related morbidity.

### 2.3. Pre-immune checkpoint inhibitors era

Prior to the advent of ICIs there were no approved therapies for advanced CSCC. Small, heterogenous retrospective analyses have suggested some activity for cytotoxic chemotherapy such as platinums, taxanes, and fluoropyrimidines [76]. However, chemotherapy is often poorly tolerated in the older populations predisposed to this malignancy and in the absence of a survival benefit it is hard to justify its use in elderly and comorbid populations. Epidermal growth factor receptor (EGFR) inhibitors have demonstrated some efficacy. A phase II study of cetuximab demonstrated responses in 28% of patients (*n* = 10/36) and in an additional study with panitumumab 31% (*n* = 5/16) of patients responded [77,78]. However, response rates are relatively low and often short-lived. In the era of ICIs, the role of these therapies is limited and usually only considered post progression on first-line ICIs.

### 2.4. Immune checkpoint inhibitors (ICI) for CSCC

Immune checkpoint molecules are the primary targets of ICIs. They regulate the balance between activating and inhibitory signals within the immune system, particularly among T cells, antigen-presenting cells (APC), and tumor. These targets include cytotoxic T-lymphocyte antigen 4 (CTLA-4, targeted by ipilimumab), programmed cell death protein 1 (PD-1, targeted by pembrolizumab, nivolumab, and cemiplimab), and programmed cell death protein ligand 1 (PD-L1, targeted by durvalumab and avelumab). In a functioning anti-tumor immune response, APCs detect tumor antigens and present them to T cells during the “priming phase” [79]. Subsequent interaction between the major histocompatibility complex (MHC) on the APC and the T cell receptor leads to T cell activation and the initiation of an anti-tumor response (i.e. the effector phase) [79]. However, T cell activation is a complex process that also requires the involvement of various co-stimulatory and inhibitory signals. As part of an inherent feedback mechanism to prevent autoimmunity, certain immune checkpoints – such as CTLA-4 and PD-1—deliver inhibitory signals that limit T cell activity. The ability of these co-inhibitory receptors to suppress T cell responses against tumors provided the rationale for developing ICIs, with the most studied targets being CTLA-4/B7 and PD-1/PD-L1.

There is excellent biologic rationale for the use of ICIs in CSCC supported by molecular, immunologic, and tumor microenvironment features. CSCC has one of the highest median TMBs of any malignancy, with 45.2 mutations per megabase [80]. This is roughly three times the median TMB of melanoma. High TMB is associated with ICI response in numerous malignancies [81] and is thought to partly explain the sensitivity of CSCC to ICIs. CSCC also commonly upregulates PD-L1 to evade immune detection and promote T cell exhaustion. PD-L1 has proven to be an effective biomarker in other malignancies, such as non-small cell lung cancer and mucosal head and neck SCC [82,83]. Other checkpoint molecules like LAG3 and TIGIT have also been identified in CSCC suggesting other potential immune targets [84,85]. CSCC also appears to be closely linked to the immune system, highlighted by a strong predisposition to its development in immunocompromised hosts. The tumor microenvironment in CSCC has emerged as a pivotal determinant in ICI response and resistance and remains a key focus of ongoing translational research. Altogether, the genetic, molecular, and immunologic landscape of CSCC provides compelling justification for the therapeutic use of ICIs.

## 3. Introduction to the compound

**Figure 1.**
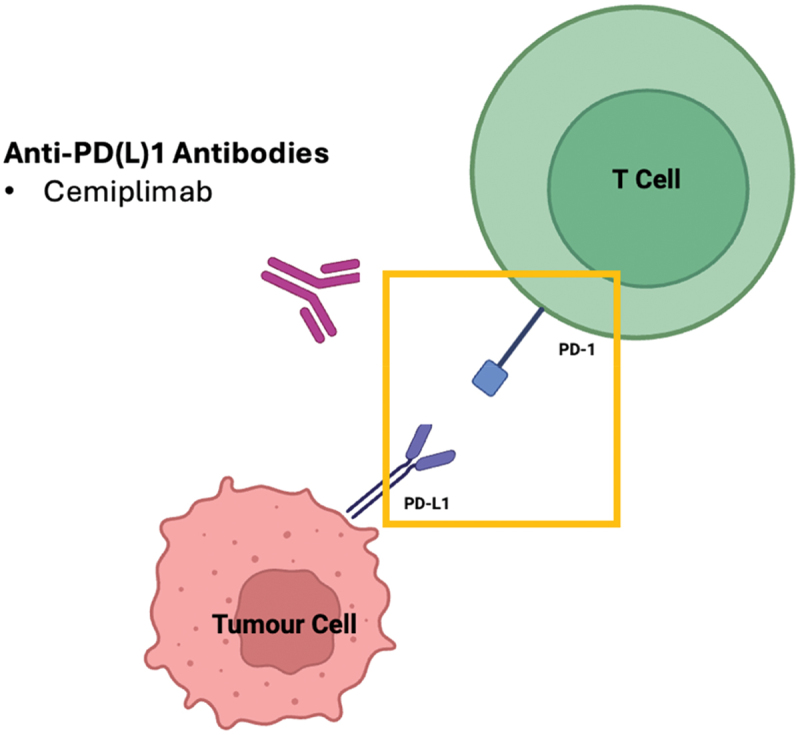
Cemiplimab blocks the interaction between PD-L1 on tumor cells and PD-1 on immune cells. *Programmed cell death protein 1 (PD-1), programmed cell death protein ligand 1 (PD-L1) (Created in BioRender. Pizzolla, A. (2026) https://BioRender.com/6lr9gon).*

Immune-checkpoint inhibitors have revolutionized the management of advanced CSCC. Cemiplimab is a high-affinity IgG4 fully human monoclonal antibody to the PD-1 receptor that blocks PD-1/PD-L1 mediated T-cell inhibition (Figure 1). Cemiplimab was the first ICI to demonstrate benefit in the advanced CSCC population. Through binding to PD-1, cemiplimab blocks its interaction with ligands, PD-L1 and PD-L2, thereby releasing the brakes on the immune system and improving T-cell mediated anti-tumor activity.

As described in detail in the sections below, cemiplimab has demonstrated excellent clinical efficacy in the management of advanced CSCC with high response rates and a potential for durable disease control [9]. It has also more recently demonstrated high major pathological response rates in the neoadjuvant setting and improved DFS in the adjuvant setting for high risk resected CSCC [11,12].

## 4. Pharmacology

Both the first-in-human (Study 1423, NCT02383212) and registrational phase II (Study 1540, NCT02760498) studies in CSCC demonstrated that cemiplimab has linear and dose proportional pharmacokinetics across the 1–10 mg/kg dose range when administered intravenously every 2 weeks [86]. Cemiplimab, being a monoclonal antibody that targets a cell surface receptor, is thought to undergo target-mediated elimination, which can become saturated at lower drug levels leading to non-linear pharmacokinetics [87]. Such linear pharmacokinetics suggested that the target-mediated pathways were saturated at the tested concentrations. Based on this, a weight-based dose of 3 mg/kg administered intravenously every 2 weeks was initially chosen as the phase II dose to achieve full target saturation across all patients [88].

However, other monoclonal anti-PD1 antibodies, such as nivolumab and pembrolizumab, had demonstrated minimal changes in clinical efficacy or safety across different drug exposure levels, indicating that variations in exposure had little impact on outcomes at the doses studied [89,90]. Additionally, monoclonal antibodies are highly target-specific, often resulting in a broader therapeutic window, and while body weight can influence the pharmacokinetics, it has been demonstrated that overall this impact is relatively minor [91]. At 1 mg/kg dosing cemiplimab demonstrated clinical efficacy and at 10 mg/kg there were no dose-limiting toxicities. This reflects the wide therapeutic window of cemiplimab. A study was then conducted to determine a fixed cemiplimab dose administered every 3 weeks that would achieve drug exposure comparable to the initial 3 mg/kg two-weekly regimen. This would facilitate better patient compliance and convenience. This occurred using population pharmacokinetic modeling based on serum concentration data from studies NCT02383212 and NCT02760498, where most patients received 3 mg/kg every 2 weeks. A fixed dose of 350 mg three-weekly was suggested to provide similar steady-state exposure to the weight-based regimen. This fixed regimen was incorporated into Group 3 of the 1540 study and exposure data was subsequently compared to the simulated exposures and what was reported with 3 mg/kg dosing [88]. Post-hoc analysis in 505 patients (weight range: 30.9–156 kg, median 76.1 kg) demonstrated steady-state exposure and variability were similar between both regimens [88]. The influence of body weight on exposure was evaluated using exposure-versus-weight plots, including data from patients at weight extremes. In this study, observed cemiplimab concentrations from 51 patients with metastatic CSCC were also overlaid on simulated concentration–time profiles from 2000 virtual patients receiving 350 mg three-weekly, confirming similarity in exposure and supporting the reliability of the model-based dose selection [88]. Key pharmacokinetic parameters from the post-hoc analysis with the final model included a volume of distribution for cemiplimab of 5.20 L (24.3% variability) and elimination half-life 19.2 days (29.5% variability). Clearance reduced by 34.6% from 0.33 L/day (40% variability) after the initial dose to 0.21 L/day (39.5% variability) at steady state [88]. Patient factors such as body weight, albumin, and IgG levels altered exposure by less than 20% and were not thought to be clinically relevant [92]. Renal and hepatic impairments have not been shown to have any impact on dosing and thus no adjustments are currently recommended in these settings [92]. These findings, along with the clinical efficacy and safety outcomes reported in the phase II clinical trial, supported the approval of the 350 mg three-weekly regimen by the United States (US) Food and Drug Administration (FDA) and European Commission for the treatment of metastatic or locally advanced CSCC not amenable to curative surgery and/or radiotherapy.

Pharmacokinetic modeling has also demonstrated that a 600-mg cemiplimab dose every 4 weeks could maintain trough levels between those seen with 3 mg/kg two-weekly and 350 mg three-weekly regimens. Thus, this dose was utilized in Group 4 of the registrational 1540 study based on its ability to achieve effective drug levels whilst remaining within a safe exposure range [93]. This regimen produced a higher average peak concentration (Cmax) than the two-weekly and three-weekly regimens. Trough levels were consistent across all groups and observed drug levels at steady state closely matched those predicted by population modeling [93].

## 5. Clinical efficacy

In the dose-escalation phase of the cemiplimab phase I trial (Study 1423, NCT02383212), a patient with advanced CSCC experienced a profound and long-lasting treatment (> 16 months) response [94]. This was the first indication of ICIs providing treatment success in advanced cases of CSCC. The results of the phase I expansion cohorts in both locally advanced or metastatic CSCC and the primary analysis of the registrational phase II trial (NCT02760498) in a metastatic cohort (Group 1) were subsequently reported in the *New England Journal of Medicine* in 2018 [9].

**Figure 2.**
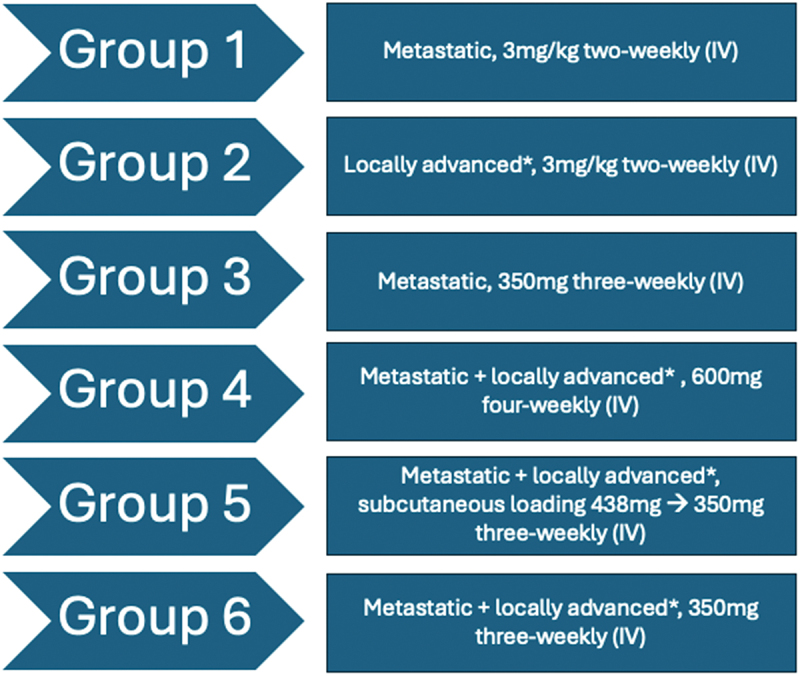
Summary of the stage of disease and cemiplimab regimen for the six trial arms in the NCT02760498 phase II clinical trial. *IV – intravenous.*

The phase II study included six cohorts of patients with advanced CSCC. Group 1 (metastatic CSCC) and Group 2 (locally advanced CSCC) received cemiplimab at 3 mg/kg 2 weekly for up to 96 weeks. Group 3, comprising patients with metastatic disease, received cemiplimab 350 mg three-weekly for up to 54 weeks. Group 4, an advanced CSCC cohort, was treated with 600 mg four-weekly for up to 48 weeks. Group 5 received a subcutaneous loading dose of cemiplimab 438 mg followed by 350 mg intravenously every 3 weeks. Finally, Group 6 received cemiplimab 350 mg three-weekly for up to 108 weeks. A summary of these cohorts can be seen in Figure 2. Patients with any nodal disease were included in the metastatic cohorts of Group 1 and 3, whilst Group 2 patients had locally advanced patients without any nodal disease.

*Locally advanced disease not amenable to curative surgery and/or radiotherapy.

In the phase I expansion cohort, the objective response rate was 50% (*n* = 13/26, 95% CI 34–61), while in the metastatic phase II cohort (Group 1), the response rate was 47% (*n* = 28/59, 95% CI 34–61) [9]. There was also suggestion of durability in response and of the 28 patients who responded to ICIs, 57% maintained their response for more than 6 months and 82% were still responding and on treatment at the time of data analysis [9]. Further, follow-up in this cohort has since demonstrated ongoing durability of response [95]. At 12 months, progression-free survival (PFS) was 53% (95% CI 37–66) and overall survival (OS) was 81% (95% CI 68–89). Subgroup analyses revealed similar efficacy between patients with regional and distant metastatic disease, with response rates of 43% and 49%, respectively.

The subsequent primary analysis of the fixed dosing regimen (350 mg three-weekly) in a metastatic CSCC cohort (Group 3) of the phase II study also demonstrated significant activity and durability of response [95]. With a median follow-up of 8.1 months (range, 0.6 to 14.1) the response rate was 41.1% (95% CI, 28.1 – 55). Durability was also demonstrated with an estimated 8-month and 12-month duration of response of 95% and 88.9%, respectively [95]. An additional cohort of both metastatic and locally advanced participants, Group 6, also received fixed dosing cemiplimab 350 mg/three-weekly. At 8.7 months of median follow-up, the response rate (*n* = 165) was 44.8% and median duration of response and PFS were not reached [96].

Patients in Group 4 (*n* = 63) received the extended dosing regimen of 600 mg cemiplimab four-weekly. With a median follow-up 22.4 months (range: 1.0–39.8), a response rate of 62% (95% CI: 48.8 – 73.9) was reported. Overall, 22% (*n* = 14) achieved a complete response and 40% (*n* = 25) a partial response to treatment. Numerically, the observed response rates were higher with this regimen compared to the two-weekly and three-weekly schedules. However, intra-cohort comparisons are limited due to differences in baseline characteristics. Notably, 86% of patients in Group 4 received cemiplimab as first-line therapy compared with 66% in Groups 1–3. Preliminary data from this prospective study indicated that fluorodeoxyglucose-(FDG)-Positron Emission Tomography (PET) was as effective as conventional imaging in identifying overall treatment responses, but in line with retrospective reports it also appeared to detect a higher proportion of complete responses [93,97].

The integrative analysis of the registrational cohorts of this key phase II study demonstrated an overall response rate of 46.1% (95% CI 38.9–53.4). The median time to complete response was 11.2 months and 87.8% of those who achieved at least a partial response had an ongoing response at 12 months [98]. With longer term follow-up, the durability of responses to cemiplimab has been well documented, as well as an increase in complete response rates over time [95,98,99]. There is an additional cohort, Group 5, who have received an initial subcutaneous loading of cemiplimab followed by intravenous treatments in whom results have not been reported.

Other ICIs have also since been explored in advanced CSCC. In the KEYNOTE-629 trial (NCT03284424) (*n* = 159) patients received pembrolizumab 200 mg three-weekly. The objective response rate was 50% (95% CI 36.1–63.9) and 35.2% (95% CI 26.2–45.2) in the locally advanced and metastatic cohorts, respectively. In both cohorts, the median duration of response was not reached at time of analysis [100]. Based on these results, pembrolizumab received FDA approval for advanced CSCC. Additionally, the CARSKIN study (NCT02883556) in untreated patients reported a 41% response rate at 15 weeks and a median OS of 25 months [101]. Similar outcomes have also been observed with nivolumab in a phase II first-line study (NCT03834233) [102]. Intralesional cemiplimab is also being evaluated in clinical trials including cemiplimab alone (NCT03889912) and cemiplimab in combination with the toll-like receptor agonist vidutolimod (NCT04916002).

Exploratory data from the phase II cohorts of the key cemiplimab clinical trials has demonstrated that although ICI responders tend to have a higher average TMB, there remains considerable overlap in TMB values between responders and non-responders [93]. Some studies have suggested that ICIs may be less effective in patients with non – head and neck primaries compared with those arising in sun-exposed head and neck sites [103]. Proposed hypotheses relate to differences in TMB or tumor microenvironment (TME) and this is an area of ongoing research. For CSCC arising in immunocompromised individuals, the current literature suggests a median TMB comparable to that seen in immunocompetent patients [104]. Nonetheless, specific alterations such as greater prevalence of HRAS mutations and a distinct mutational signature related to azathioprine exposure are reported [104,105]. This indicates that immunosuppression may shape the mutational evolution of advanced CSCC differently from classical UV-induced tumorigenesis [106]. Since TMB is a surrogate for neoantigen load, emerging tools like the agretopicity index – which also integrates mutation type, gene expression, and MHC binding – may offer superior prediction in the future [107–109]. Similarly, analyses of PD-L1 expression – measured as the percentage of tumor cells exhibiting membranous PD-L1 staining (22C3 assay – Dako, Agilent, Santa Clara, CA, USA) – have revealed that treatment response is independent of PD-L1 status [93,99].

The TME is increasingly recognized as another key determinant of response and resistance to ICIs. Three broad classifications of the TME have been proposed including immune-inflamed, immune-excluded, and immune-desert [110]. Immune-inflamed has been mostly strongly correlated with ICI response in other malignancies [111]. Gene expression profiling allows an assessment of the expression of thousands of genes in a cell population at a certain time and has revealed distinct expression clusters between ICI-responsive and resistant tumors, potentially serving as a surrogate for the inflammatory state of the TME. For example, signatures reflecting activity in the interferon-gamma pathway have been shown to correlate with ICI response in melanoma. Most notably, the T cell-inflamed gene expression profile (TcellGEP) signature incorporates 18 genes encompassing interferon gamma, antigen presentation, chemokine expression, cytotoxicity, and adaptive immune resistance [112]. In the KEYNOTE-629 trial of pembrolizumab in advanced CSCC, the presence of the TcellGEP signature was linked to higher tumor response rates [113]. Future research should focus on the prospective collection of large, well-annotated cohorts of ICI responsive and resistant CSCC samples for comprehensive analyses. This should include systematic evaluation of candidate biomarkers, such as TMB, immune checkpoints, tumor-infiltrating lymphocytes, and gene expression signatures from bulk profiling. Emerging approaches that integrate spatial context with transcriptomic data will be essential in better understanding how these factors shape ICI response.

Host factors are also thought to influence response to ICIs. Germline genetic variation, including human leukocyte antigen (HLA) diversity and polymorphisms in immune-related genes such as *JAK – STAT* family members or *NF-κB*, has been associated with reduced ICI efficacy [114,115]. The gut microbiome also plays a critical role in modulating both innate and adaptive immune responses, with several studies demonstrating its impact on ICI outcomes. For instance, enrichment with *Faecalibacterium* species has been linked to higher immune cell infiltration, while *Bifidobacterium* species have been shown to enhance ICI activity in murine models [116,117]. The gut microbiome is highly dynamic and influenced by factors such as infection, diet, and medications such as proton-pump inhibitors and antibiotics which can alter microbial composition and diversity. Identifying consistent microbial biomarkers predictive of response remains an active area of investigation and is not well explored in advanced CSCC.

Further research is also warranted in exploring the optimal duration of ICIs in advanced CSCC. Clinical trials have generally administered ICIs for up to 2 years in responding patients, though prolonged treatment can be burdensome for older individuals and may increase the risk of cumulative toxicity. Evidence from metastatic melanoma suggests that extended therapy may not be necessary for durable responses [118,119], with studies such as KEYNOTE-006 demonstrating sustained benefit after treatment cessation at 2 years, and there are ongoing trials exploring earlier discontinuation following response (NCT02821013, NTR7502). Similar findings are emerging in advanced CSCC but data are overall limited. One retrospective series of 95 patients treated with cemiplimab found that 23% (*n* = 22) discontinued therapy early (due to toxicity, comorbidity, compliance/patient preference, or complete response) without compromising outcomes [120]. In the pooled phase II cemiplimab studies, the median time to complete response was 11.2 months, and 87.8% of responders maintained benefit at 12 months [98]. Prospective studies are needed to define the minimum effective duration of ICI therapy, with response-adapted or fixed shorter-course strategies offering potential to minimize toxicity and treatment burden, particularly in older patients.

### 5.1. Data in adjuvant setting

Surgery is standard of care for resectable CSCC, achieving cure in 95% of cases [121,122]. However, there remains a subgroup of patients who experience disease recurrence post-curative surgery and adjuvant radiotherapy. The randomized phase III Postoperative Skin Trial/Trans-Tasman Radiation Oncology Group [POST/TROG] 05.010 study (NCT00193895) found no additional benefit of adding carboplatin to adjuvant radiotherapy in patients at elevated risk of recurrent CSCC [69]. This study randomly assigned 321 patients to adjuvant radiotherapy alone or radiotherapy and weekly carboplatin (area under the curve 2). There were no significant differences seen in DFS or OS [69] A subsequent recursive partitioning analysis has, however, identified a particularly high-risk population in this cohort consisting of those with extranodal extension and nodal size greater than 22 mm [123].

The C-POST trial (NCT03969004) was a randomized phase III study (*n* = 415) evaluating patients at high risk of CSCC recurrence following surgical resection and postoperative radiotherapy [12]. Participants were randomized 1:1 to receive either cemiplimab or placebo for 12 months. Cemiplimab was administered at 350 mg three-weekly for 12 weeks, followed by 700 mg six-weekly for up to 36 weeks (prior to an amendment patients received the three-weekly schedule for 48 weeks). High-risk disease was defined by nodal features (extracapsular extension with the largest node >20 mm or ≥3 involved nodes) or non-nodal features such as in-transit metastases, T4 lesions with bone invasion, perineural invasion, or locally recurrent tumors with more than one additional risk factor. The primary endpoint was DFS. Participants in this study were typical of the CSCC population with a median age of 71 years, 83.9% were male, and in 82.7% of cases the primary CSCC lesion was from the head and neck. Overall, 58.3% (*n* = 242) had high risk nodal disease, with extracapsular extension being the most common characteristic. Demographics were well balanced across both treatment arms. With a median follow-up of 24 months, cemiplimab significantly improved DFS compared to placebo (HR 0.32, 95% CI 0.20–0.51, *p* < 0.001), with estimated 24-month DFS rates of 87.1% and 64.1%, respectively. Most CSCC recurrences occurred within the first-year post-surgery and radiotherapy, consistent with the disease’s natural history. In the C-POST trial, DFS curves separated early and remained separated, indicating durable benefit with adjuvant cemiplimab. Locoregional recurrences were more frequent than distant ones, with cemiplimab reducing this risk by 80% and the risk of distant recurrence by 65%. In an exploratory analysis, DFS benefit was seen with both the three-weekly schedule for 48 weeks and the three-weekly for 12 weeks then switching to the six-weekly schedule. The safety profile aligned with previous studies using cemiplimab, with 9.8% discontinuing due to adverse events and only one treatment-related death. Quality of life was generally preserved [12]. While overall survival benefit is not yet confirmed, likely due to cross over allowing cemiplimab use on recurrence, reducing early recurrence remains a meaningful clinical advantage in this high-risk population.

The phase III KEYNOTE-630 (NCT03833167) evaluated adjuvant pembrolizumab in 450 patients with resected, high-risk locally advanced CSCC who had completed post-operative radiotherapy and has only been reported in preliminary form [124]. At a median follow-up of 28.6 months, pembrolizumab did not significantly improve recurrence-free survival (RFS) compared with placebo (24-month RFS 78.3% vs 68.6%, HR 0.76, *p* = 0.072). Treatment-related adverse events were more frequent with pembrolizumab (63.8% vs 41.1%); however, there were no treatment-related deaths. A high number of deaths unrelated to disease progression were observed and may have offset the reduction in recurrences. The trial stopped early due to futility [124]. This study is contrasted with the C-POST trial in Table 1.

**Table 1.** Summary of the key features and findings from the C-Post and KEYNOTE-630 adjuvant clinical trials in high-risk resected cutaneous squamous cell carcinoma (CSCC).

	C-POST clinical trial [11]	KEYNOTE-630 [106]
Sample size, n	415	450
Randomisation	1:1 Cemiplimab 350 mg three-weekly for 12 weeks then 700 mg six-weekly up to 36 weeks vs placebo	1:1 Pembrolizumab 400 mg six-weekly for up to 9 cycles (approx. 12 months) vs placebo
Definition of high-risk disease	**Nodal** ECE with ≥1 node ≥20 mm ≥3 nodes regardless of ECE **Non-nodal** Skin/subcutaneous metastases > 20 mm from the primary lesion but not beyond the regional nodal basin Clinical and/or radiologic involvement of named nerves Invasion of cortical bone or skull base Recurrent CSCC that arises within the area of previously resected tumor plus ≥1 additional high-risk feature^#^	**Nodal** ECE with ≥1 node >2 cm ≥2 nodes involved **Non-nodal** Any gross cortical bone, skull base and/or skull base foramen invasion Any index tumor with ≥2 of: tumor ≥4 cm with >6 mm depth of invasion beyond subcutaneous fat perineural invasion for nerve <0.1 mm (≥3 foci) or any involved nerve ≥0.1 mm in diameter poor differentiation and/or sarcomatoid and/or spindle cell histology recurrent disease or satellite lesions and/or in-transit metastases lymphatic or vascular involvement
Primary endpoint	Disease free survival (DFS)	Recurrence free survival (RFS)
Median follow up	24 months	28.6 months
Primary outcome	DFS benefit met: HR 0.32 (95%CI 0.20 – 0.51, *p* < 0.001)	RFS not significant: HR 0.76 (95%CI 0.53 -1.10, *p* = 0.072)
24 months DFS/RFS rate	Cemiplimab: 87.1% vs Placebo: 64.1%	Pembrolizumab: 78.3% vs Placebo: 68.6%
Overall survival (OS)	Ongoing, not mature HR 0.78 (95%CI 0.39 – 1.56)	No OS benefit: HR 1.47 (95%CI 0.87 – 2.48)
Adverse events (Grade 3+)	Cemiplimab: 23.9% vs Placebo: 14.2%	Pembrolizumab: 26.3 vs Placebo: 16.5%

^#^other high risk features included: ≥N2b disease associated with recurrent lesion, nominal ≥T3 or poorly differentiated histology and recurrent lesion ≥20 mm diameter.

Abbreviations: ECE – extracapsular extension, CSCC – cutaneous squamous cell carcinoma, CI – confidence interval.

The results of the recursive partitioning analysis of the POST trial, in addition to highlighting a high-risk group, also identified that other groups included experienced high DFS rates following standard surgery and radiotherapy. The C-POST trial was restricted to a population that remained at high risk of recurrence after standard treatment and were most likely to benefit from an effective adjuvant treatment. It is important to note that C-POST and KEYNOTE-630 did differ in their inclusion criteria in how they defined their “high-risk” population, with some lower risk patients being eligible for KEYNOTE-630 but not C-POST.

### 5.2. Data in neoadjuvant setting

The success of ICIs in the advanced and adjuvant settings has inevitably generated interest in neoadjuvant approaches. In melanoma, neoadjuvant approaches have demonstrated superiority in EFS compared with adjuvant approaches and have also offered the potential to tailor patient treatments to pathological response [125]. There is also biological rationale for such approaches with pre-clinical data demonstrating greater expansion of tumor-resident T cell clones when ICIs are administered with tumor *in situ* [126]. With neoadjuvant pathways proving a success, most notably in locally advanced melanoma [125], there has been strong interest in exploring this paradigm in CSCC.

In a pilot trial of 20 patients with locoregionally advanced CSCC, a complete pathologic response (pCR) rate of 55% was achieved with two cycles of cemiplimab [127]. This led to a pivotal multicenter phase II study (NCT04154943) (*n* = 79) investigating up to four cycles of 350 mg three-weekly neoadjuvant cemiplimab before surgery in patients with clinical stage II-IV (M0) CSCC. This study reported a high pCR rate of 51% (95% CI 39 – 62) and a major pathological response (defined as <10% viable tumor) in a further 13% (95% CI 6 – 22) of patients [11]. A recently reported translational trial of neoadjuvant cemiplimab in resectable stage II-IV CSCC in 11 patients reported a 73% pCR rate [128]. The phase II De-Squamate trial (NCT05025813) assessed four cycles of pembrolizumab followed by risk-adapted surgical de-escalation/omission [129]. The primary endpoint was clinical or pathological complete response (cpCR). A cpCR was reported in 63% (*n* = 17) of patients and consisted of four pCRs and 13 complete clinical responses. Importantly, none of the individuals who achieved either a complete or major pathological response experienced disease recurrence during at least 6 months of follow-up [129].

Based on the phase II data, neoadjuvant cemiplimab is now being explored in a phase III clinical trial. The NRG-HN014 study (NCT06568172) is currently randomizing patients with resectable stage III-IV CSCC to standard of care surgery and adjuvant radiotherapy (if indicated) versus neoadjuvant cemiplimab (350 mg three-weekly) for four cycles followed by response adapted surgery, adjuvant radiotherapy (if indicated) and adjuvant cemiplimab pending pathological response. Patients who achieve a pCR will not receive adjuvant ICI or radiation.

Neoadjuvant approaches are of considerable interest. By achieving disease control earlier, this may reduce the need for extensive surgery or adjuvant radiotherapy. In patients who respond, they would only receive four cycles of cemiplimab, compared to 12 months in the CPOST adjuvant regimen. However, the neoadjuvant approach results in a much broader population receiving cemiplimab, whereas the adjuvant approach restricts treatment to the subgroup of patients with identified high-risk features following surgery. Neoadjuvant therapy does provide early insight into treatment response, with pathological findings enabling more individualized and informed discussions about prognosis and subsequent management.

## 6. Real-world evidence

Advanced CSCC is common in populations who are less likely to meet the stringent inclusion criteria of clinical trials. This encompasses patients who are elderly, with higher Charlson comorbidity index scores and a poorer performance status, and immunocompromised patients, such as those with solid organ transplants or hematological malignancies. This has resulted in a drive to review real-world data on both the safety and clinical efficacy of cemiplimab in these trial-ineligible populations.

Cutaneous squamous cell carcinoma is more common with increasing age. Thus, it is no surprise that in the key cemiplimab registrational trial, the median age of participants was 71 years, with 73% of participants being over 65 years of age [9]. Older patients often have a reduced functional reserve, increased frailty, and poorer performance status which can limit their eligibility for clinical trials. Whilst chemotherapy has traditionally been poorly tolerated in this population, there has been interest in reviewing the efficacy and tolerability of ICIs in elderly populations. An Australian series reported on 53 patients aged over 70 years (median 82 years) treated with anti-PD1 ICIs (51 with cemiplimab, two pembrolizumab) and identified a similar efficacy (ORR 57%, 12 month OS 63%) and acceptable safety profile to what has been reported in clinical trial populations [10]. An additional retrospective series from Italy, in 20 elderly patients with advanced CSCC, reported a response rate of 76.7% and median PFS and OS of 16 and 18 months, respectively [130]. There has also been a growing interest in the use of ICIs, such as cemiplimab, in patients in whom curative surgery may be morbid or contraindicated due to comorbidities. Orbital involvement, for example, is not uncommon in CSCC of the head and neck. Increasingly, ICIs are being reported as a successful orbital-sparing approach in cases of locally advanced CSCC where surgical cure would otherwise require orbital exenteration [131,132].

The key registrational ICI trials in advanced CSCC also excluded immunocompromised populations. A large multicentre Australian series reported on the outcomes of 286 people with advanced CSCC who were treated via a compassionate access scheme [126]. 78% of this cohort were deemed ineligible for the registrational phase I/II cemiplimab study. In this population, 31% (*n* = 88) were immunocompromised. The ORR was 51% in the immunocompromised versus 64% in the immunocompetent. Whilst multivariate analyses did identify immune status to be prognostic for both OS and PFS, the authors still reported the potential for durable and complete responses in this population albeit at a lower rate than in their immunocompetent counterparts [126]. Conversely, a smaller review from the French access program, which included 59 immunocompromised patients, found no differences in response rates, OS, or PFS compared to immunocompetent patients [103]. They did, however, identify poorer ECOG performance status (≥2) was associated with reduced PFS and OS during the first 6 months of treatment and an association between head and neck primary lesion (compared with non-head and neck) and better PFS [103]. An American retrospective series of 622 patients (133 immunocompromised) also identified no differences in OS between immunocompetent and immunocompromised patients in multivariate analysis [133]. However, this study was limited by the absence of data on disease progression and toxicity, preventing calculation of PFS and assessment of treatment-related adverse events.

The solid organ transplant populations are a particularly vulnerable cohort who face a high risk of advanced CSCC and the very real concern of allograft rejection with ICIs. Limited retrospective reports have estimated rejection rates around 40% with the use of ICIs; however, high-quality prospective data is lacking [134,135]. Historical practices of reducing immunosuppression at the time of ICI introduction and also a lack of uniformity in the management of rejection have been significant confounders. A prospective phase I study led by Carroll et al. (ANZCTR12617000741381) utilized Nivolumab in kidney transplant recipients with advanced cancer and mandated the maintenance of a patient’s immunosuppression at the time of ICI introduction [136]. This study reported a lower rejection rate (12%, 2/17) with efficacy (53% response rate, 9/17) [136]. The prospective CONTRAC-1 study (NCT04339062) focused on advanced CSCC patients with a renal allograft who received cemiplimab. This study introduced a standardized approach to immunosuppression with dynamic prednisolone dosing and mTOR inhibition [137]. In this study there were no cases of allograft rejection reported and the response rate was 46% (5/11) [137]. This contrasts with findings from another study involving eight renal transplant recipients with advanced cutaneous malignancies (five who had CSCC), which reported low-dose tacrolimus and prednisolone did not adequately prevent allograft rejection and appeared to impair responses to nivolumab with or without ipilimumab [138].

## 7. Safety and tolerability

Cemiplimab has demonstrated a favorable safety profile in both clinical trial settings and real-world populations, with no unexpected toxicities reported. Across clinical trials, treatment-related adverse events have proven manageable, treatment-related mortality has been rare, and discontinuation rates have been acceptable. Treatment-related adverse events commonly reported across studies include diarrhea, pruritus, and fatigue. Immune-related adverse events are the principal toxicities of concern. ICIs, such as cemiplimab, function by blocking inhibitory pathways (i.e PD-1/PD-L1) thereby enhancing T-cell activation and restoring anti-tumor immune responses. This can increase the risk of off-target inflammation in healthy tissue and can manifest in any organ system. The integrated analysis of Groups 1–3 of the 1540 study demonstrated 57 patients (29.5%) experienced at least one immune-related adverse event of any grade [98]. Eighteen (9.3%) patients experienced a grade 3 or higher immune-related toxicity of which the most common were pneumonitis (*n* = 5), hepatitis (*n* = 2), and diarrhea (*n* = 2) [98]. A summary of the safety outcomes from key clinical trials across advanced, neoadjuvant, and adjuvant settings is presented in Table 2.

**Table 2.** Safety and tolerability of cemiplimab across the key advanced, neoadjuvant, and adjuvant clinical trials in cutaneous squamous cell carcinoma (CSCC) to date.

	Cemiplimab dosing/frequency	Most common adverse events	Grade 3+ adverse events % (n)	Event that led to discontinuation of treatment n (%)	Event that led to death n (%)
**Advanced**					
Migden et al., 2018 (*n* = 59) (Phase II – Group 1) [8]	3 mg/kg two-weekly	Diarrhea 27% Fatigue 24% Nausea 17% Constipation 15% Rash 15%	42% (25)	5% (3)	5% (3))
Rischin et al., 2021 (*n* = 193) (Phase II – Group 1–3, integrative analysis) [95]	Group 1–2 3 mg/kg two-weekly Group 3350 mg three-weekly	Fatigue 34.7%, diarrhea 27.5%, nausea 23.8%, pruritus 21.2%, cough 16.6%	48.7% (94)	9.8% (19)	NR
Rischin et al., 2020 (*n* = 56) (Phase II – Group 3) [92]	350 mg three-weekly	Fatigue 28.6%, diarrhea 17.9%, nausea 17.9%, rash 16.1% constipation 12.5%	39.3% (22)	5.4% (3)	1.8% (1)
Rischin et al., 2024 (*n* = 63) (Phase II – Group 4) [90]	600 mg four-weekly	Diarrhea 27%, pruritus 25%, fatigue 22%, constipation 22%, rash 19%, arthralgia 19%	54% (34)	18% (11)	10% (6)
Hughes et al., 2025 (*n* = 165) (Phase II – Group 6) [93]	350 mg three-weekly	Fatigue 26.1%, diarrhea 21.2%, pruritus 21.2%, nausea 17%, asthenia 13.9%	45.5% (75)	13.9% (23)	8.5% (14)
**Neoadjuvant**					
Gross et al., 2022 (*n* = 79) (Phase II) [10]	350 mg three-weekly	Fatigue 30%, diarrhea 14%, nausea 14%, rash 14%, constipation 11%	18% (14)	1% (1)	5% (4)
**Adjuvant**					
Rischin et al., 2025 (*n* = 415) (Phase III) [11]	350 mg three-weekly for 12 weeks, followed by 700 mg every six weeks for up to 36 weeks	Fatigue 22% vs 21.6%, pruritus 16.1% vs 12.3% rash 16.1% vs 8.8% diarrhea 15.6% vs 18.6%	23.9% (*n* = 49) cemiplimab vs 14.2% (*n* = 29) placebo	9.8% cemiplimab (*n* = 20) vs 1.5% (*n* = 3) placebo	2 (1%)

Advanced CSCC is more common in older patients and in this population, there is often concern that the risk–benefit ratio for systemic therapy may be less favorable. Chemotherapy, for instance, is frequently poorly tolerated in elderly patients due to competing comorbidities and diminished physiological reserve. Historically, patients aged ≥80 years have comprised only about 4% of clinical trial participants [139,140]. However, given the predisposition for CSCC in older patients, the pivotal cemiplimab registrational trial reported a median cohort age of 71 years [9]. This has generated interest in reviewing real-world outcomes of ICI use in older populations with advanced CSCC. Current reports, whilst limited by small sample sizes, suggest similar toxicity and efficacy outcomes to what has been reported in clinical trial populations. An Australian retrospective study in patients aged over 70 years with advanced CSCC (*n* = 53) reported only two cases of grade 3 or higher immune-related adverse events and no treatment-related deaths [10]. Similarly, an Italian study (*n* = 30, median age 81 years) observed low toxicity rates, with the most common adverse events being fatigue (23.3%) and skin toxicity (33.3%) [141]. The safety of ICIs in solid organ transplant recipients also remains an area of active investigation. Recent prospective studies in this cohort have reported significantly lower rates of graft rejection compared to earlier retrospective analyses, renewing interest in this high-risk population [136,137].

## 8. Regulatory affairs

Intravenous cemiplimab 350 mg three weekly is approved for the first line treatment of metastatic or locally advanced CSCC in patients not eligible for curative surgery or radiation in the US (FDA, 2018), Europe (EMA, 2019), and Australia (TGA, 2020). Adjuvant cemiplimab is also approved in the US (FDA, Oct 2025) and Europe (EMA, Nov 2025). Cemiplimab is not currently approved for use in the neoadjuvant setting.

## 9. Conclusion

Cemiplimab, an anti-PD1 antibody, is the standard of care for patients with locally advanced or metastatic CSCC who are not candidates for curative surgery or radiotherapy. Clinical trials and real-world data consistently demonstrate its efficacy for advanced disease with a manageable safety profile comparable to other ICIs. There have been no unexpected treatment-related adverse events and discontinuation rates from toxicity are generally low. Special populations, such as organ transplant recipients, represent an area of active investigation. Emerging evidence suggests a role for cemiplimab in earlier disease settings, with promising results from a phase III adjuvant and phase II neoadjuvant studies, suggesting potential for future approval in these contexts.

## Article highlights

- Cemiplimab is a human IgG4 monoclonal immune checkpoint inhibitor (ICI) antibody targeting the PD-1 receptor.
- Cemiplimab has demonstrated a response rate of approximately 50% with a potential for durable disease control in patients with metastatic and/or unresectable locally advanced cutaneous squamous cell carcinoma (CSCC).
- Cemiplimab 350 mg every 3 weeks is the standard of care for metastatic and/or unresectable advanced CSCC.
- Cemiplimab has demonstrated high pathological complete response rates (51%) in a phase II neoadjuvant clinical trial for resectable stage II-IV CSCC.
- 12 months of adjuvant cemiplimab in high-risk resected CSCC significantly improved disease-free survival (DFS) compared to placebo (HR 0.32, 95% CI 0.20–0.51) in the C-POST trial.
- Adjuvant cemiplimab is a potential new standard of care option for patients with high-risk resected locoregionally advanced CSCC.
- Cemiplimab is well tolerated with a toxicity profile similar to other anti-PD1 ICIs.
